# School Absenteeism Among Racially and Ethnically Minoritized Transgender High School Students and Their Peers: A Cross-Sectional Study

**DOI:** 10.1016/j.jadohealth.2024.06.028

**Published:** 2024-08-15

**Authors:** Anita V. Chaphekar, Jae Sevelius, Dave Glidden, Stanley R. Vance

**Affiliations:** a Department of Pediatrics, University of California, San Francisco, San Francisco, California; b Department of Psychiatry, Columbia University, New York, New York; c Department of Epidemiology and Biostatistics, University of California San Francisco, San Francisco, California

**Keywords:** Absenteeism, High school, Racially and ethnically minoritized groups, Transgender

## Abstract

**Purpose:**

To explore absenteeism among racially and ethnically minoritized transgender youth (trans REMY) compared to their White transgender (trans WY) and racially and ethnically minoritized cisgender (cis REMY) peers and identify associated psychosocial factors.

**Methods:**

Biennial California Healthy Kids Survey 2017-2019 data was analyzed with a weighted sample of California’s secondary school population. Students reported past 30-day absences due to mental health and harassment, depressive symptoms, suicidal ideation, cyberbullying, victimization, and school connectedness. Poisson and linear regression compared absenteeism and psychosocial factors among peer groups. For trans REMY, Poisson regression assessed associations between absenteeism and psychosocial factors. Analyses were adjusted for grade, sex, and socioeconomic status.

**Results:**

The analytical sample (n = 25,085) included 206 trans REMY, 64 trans WY, and 24,815 cis REMY. Trans REMY had higher relative risk of absenteeism due to mental health concerns and harassment compared to cis REMY (adjusted relative risk 2.9, 95% confidence interval 2.1–4.0 and adjusted relative risk 8.1, 95% confidence interval 4.0–16.6, respectively) but similar risk when compared to trans WY. For trans REMY, depressive symptoms, suicidal ideation, and victimization were associated with higher relative risk of absenteeism due to mental health concerns. Cyberbullying was associated with a higher risk of absenteeism due to harassment. Higher school connectedness was associated with lower risk of absenteeism due to mental health concerns.

**Discussion:**

Trans REMY reported higher rates of school absenteeism due to mental health concerns and harassment compared to some of their peers. Mental health symptoms, victimization, cyberbullying, and school connectedness were associated with absenteeism among trans REMY.

For adolescents, school is a critical environment that impacts future career pursuits and promotes development into adulthood [1,2]. School absenteeism is associated with several negative outcomes, including mood disorders, decreased graduation rates, future unemployment, increased risk for substance use, and negative health outcomes [3–6]. Given these negative associations with absenteeism, it is a public health concern that school absence rates have been increasing in recent years, particularly among high school students [7].

School absenteeism may disproportionately impact minoritized youth populations. Gender minority stress theory [8] asserts that minoritized youth experience societal stressors specific to their marginalized identities, and internalization of these stressors increases their susceptibility to poor psychosocial outcomes, including school absenteeism. For example, compared to White peers, racially and ethnically minoritized youth (REMY) have higher rates of absenteeism [7]. Moreover, compared to cisgender peers, transgender youth have higher rates of absenteeism with risk factors that include discrimination and victimization [9,10]. Gender minority stress theory [8] can help conceptualize how racially and ethnically minoritized transgender youth (trans REMY) may be uniquely vulnerable to school absenteeism due to social stressors related to experiences of being both gender expansive and racially and ethnically minoritized. However, there is a lack of studies that explore disparities in absenteeism experienced by these youth.

Protective and risk factors for absenteeism have not been examined specifically among trans REMY, but studies with other youth populations may indicate potential factors. Bullying is associated with absenteeism among general transgender youth samples [9,10]. Students who feel unsafe or threatened at school may avoid school with deleterious impacts on their academic futures [11,12]. Depression and suicidality are also associated with school absenteeism [4,6]. Transgender youth have staggering rates of depression and suicidality compared to cisgender peers [13–15]. Similarly, a steep increase in suicide death rates is documented among REMY compared with White peers [16,17]. In terms of potential protective factors, school connectedness—a student’s sense of belonging to school, sharing it’s values, and feeling teachers care—is associated with better mental health and less risky behaviors among students [10,18,19]. Accordingly, school connectedness should be explored as a possible protective factor against absenteeism among trans REMY.

Given the paucity of literature on absenteeism among trans REMY, we aimed to explore school absenteeism due to mental health concerns and harassment among trans REMY using a large, statewide school-based dataset. We compare their absenteeism with two peer groups: White transgender youth (trans WY) and racially and ethnically minoritized cisgender youth (cis REMY). These peer groups were chosen because trans REMY share at least one minoritized identity with trans WY (being gender expansive) and cis REMY (being racially and ethnically minoritized). Comparing students who share at least one minoritized identity based on race, ethnicity, or gender identity could provide insight into the unique risks that trans REMY face for school absenteeism. We also explore potential protective and risk factors for absenteeism among trans REMY by examining associations between absenteeism and various psychosocial factors such as school connectedness, depression, suicidal ideation, victimization, and cyberbullying. Informed by the gender minority stress theory [8], we hypothesize that trans REMY will report higher rates of absenteeism compared with trans WY and cis REMY. We also posit that among trans REMY, absenteeism will be associated with school-based psychosocial factors. By documenting absenteeism among trans REMY and school-based protective and risk factors, we can elucidate targets that can be addressed by supports to promote their school attendance.

## Methods

### Data source

Data were utilized from 9th and 11th grade students who participated in the Biennial State California Healthy Kid Survey (CHKS) administered from Fall of 2017 to Spring of 2019. The CHKS is administered to high school students who are in the 9th or 11th grade. With support from the California Department of Education and California Department of Health Care Services, CHKS is administered biennially by WestEd. The survey is anonymous and a modular, self-reported assessment of school connectedness, school climate, school safety, well-being, and resilience factors [20]. 109 California schools were randomly selected to participate. Student assent and opt-out parental consent were required for survey participation per State law [20]. The average response rate was 75%. Of all respondents, 1% were excluded due to missing data in required modules, and an additional 1% were excluded due to response validity concerns. The response data were weighted to reflect the secondary school student population of California. The initial survey sample included 34,312 high school students. The California Office of Statewide Health Planning and Development Committee for the Protection of Human Subjects approved survey implementation. The University of California, San Francisco, Institutional Review Board approved this study. The data that support the findings of this study are available from WestEd under contract to the California Department of Education. Restrictions apply to the availability of these data, which were used under license for this study. Data are available from WestEd with their permission at www.wested.org.

## Study Measures

### Demographics

Gender identity was assessed with the survey question “Are you transgender?” with these possible responses: “No, I am not transgender,” “Yes, I am transgender,” “I am not sure if I am transgender,” and “Decline to Respond.” Those who answered “no” were categorized as cisgender and “yes” as transgender. Those who responded “I am not sure if I am transgender” or “Decline to Respond” were excluded from analyses.

Race was assessed with the survey question: “What is your race?” (options were American Indian or Alaska Native, Asian, Black or African American, Native Hawaiian or Pacific Islander, White, or Mixed/two or more races). Ethnicity was assessed with the question: “Are you of Hispanic or Latino origin?” (yes/no). Students were categorized as REMY if they selected Hispanic or Latino origin or a race other than White. Notably, students who selected Hispanic or Latino origin and selected White were categorized as REMY. For this analysis, students were grouped into the following peer groups based on their responses to race, ethnicity, and gender identity: trans REMY, trans WY, and cis REMY.

Students also indicated their school grade and receipt of free or reduced-priced lunch, which was used in this analysis as a proxy for socioeconomic status. Sex was assessed with the question: “What is your sex?”. Notably, the survey did not query students on their sex assigned at birth or make a distinction between birth-assigned sex and gender identity.

### Outcomes

School absenteeism was assessed with the following CHKS question: “In the past 30 days, did you miss a day of school for any of the following reasons? (Mark all that apply).” For this study, responses of interest included: “being bullied or mistreated at school” and “felt very sad, anxious, stressed, or angry.” Students who marked “being bullied or mistreated at school” were characterized as reporting absenteeism due to harassment; those that marked “felt very sad, anxious, stressed, or angry” were characterized as reporting absenteeism due to a mental health concern.

### Psychosocial factors

We explored depressive symptoms, suicidal ideation, cyberbullying, and victimization as potential risk factors for absenteeism. Past-year depressive symptoms were assessed with the question, “During the past 12 months, did you ever feel so sad or hopeless almost every day for two weeks or more that you stopped doing some usual activities?” (yes/no). Suicidal ideation was assessed with the question, “During the past 12 months, did you ever seriously consider attempting suicide?” (yes/no). Cyberbullying was assessed with the question, “During the past 12 months, how many times did other students spread mean rumors or lies, or hurtful pictures about you online, on social media, or on a cell phone?” Responses to cyberbullying were dichotomized to 0 times or never or one or more times. School-based victimization was assessed by a nine-item measure asking how many times (0, 1, 2–3, or four or more times) a student experienced physical or verbal bullying on school property in the past 12 months. Example items included: “been pushed, shoved, slapped, hit, or kicked by someone who was not just kidding around” and “had mean rumors or lies spread about you.” Responses to victimization were dichotomized to 0 times or one or more times.

We explored school connectedness as a potential protective factor against absenteeism. School connectedness was assessed with a five-item measure, with statements such as “I am happy to be at this school.” Likert-scale response options ranged from 1- “strongly disagree” to 5- “strongly agree.” Total scores for school connectedness were created by summing Likert scores for each item, with higher total scores indicating higher level of school connectedness.

### Statistical analysis

Analyses were performed using STATA 17.1 (Stata Corp, College Station, Texas), and the sampling plan and weights were provided by WestEd using the STATA svy package. Descriptive analyses were conducted for demographic variables, types of absenteeism, and other psychosocial factors. Given the weighted sample, estimated prevalence was calculated for dichotomous measures, and the estimated mean was calculated for school connectedness. The demographic differences among peer groups were evaluated using chi-square tests. Poisson regression with a log link and a robust variance were used to derive relative risk for absenteeism, depression, suicidality, cyberbullying, and victimization comparing peer groups [21]. Linear regression was used to model means of school connectedness as a function of peer group. For school connectedness, we used the raw scores and standardized scores by dividing the measure by the standard deviation. To address the discreteness of absenteeism and psychosocial factors among trans REMY, we utilized Poisson regression with an identity link and a robust variance. We modeled each form of absenteeism as a function of each psychosocial factor. All analyses were run as unadjusted analyses and adjusted for grade, sex, and receipt of free or reduced-price lunch (proxy for socioeconomic status), as these are established covariates in studies examining absenteeism, mental health symptoms, and other psychosocial factors among transgender youth [10,15,22].

All hypothesis testing used 2-tailed *p* < 0.05 as the significance level and 95% confidence intervals (CI). Data were missing for depression (0.9%), suicidality (0.7%), cyberbullying (7.5%), victimization (2.6%), school connectedness(2.8%), reported sex (17.9%), race (12.5%), ethnicity (0.3%), and receipt of free lunch (0.6%). Missing data were deleted list-wise for all analyses.

## Results

The survey sample included 34, 312 high school students. Data were excluded from students who responded “I am not sure” or “Decline to respond” to the gender identity question (n = 3,786), declined to answer the race and ethnicity questions (n = 275), and were White and not transgender (n = 5,279). Data could only be excluded once; this is pertinent for students with multiple reasons for their data being excluded. The final analytical sample comprised data from 25,085 students. Of the analytical sample, 0.8% identified as transgender (n = 206), and 74% of transgender students identified as being REMY. Table 1 shows the demographic characteristics of each peer group. There was a significant difference in the estimated proportions of races between the trans REMY and cis REMY groups (*p* = .01), and a significant difference in the estimated prevalence of receiving reduced or no-cost lunch among the three cohorts (*p* < .001).

Table 2 provides estimated prevalence of types of absenteeism, depressive symptoms, suicidal ideation, cyberbullying, and victimization for each peer group. Additionally, Table 2 shows estimated raw and standardized means for school connectedness.

Absenteeism due to mental health concerns and absenteeism due to harassment were compared between peer groups (Table 3). Compared to cis REMY, trans REMY had higher adjusted relative risk (ARR) of absenteeism due to mental health concerns (ARR 2.9, 95% CI 2.1–4.0) and absenteeism due to harassment (ARR 8.1, 95% CI 4.0–16.6). There were no significant differences in risk for absenteeism between trans REMY and trans WY.

Table 3 shows the comparisons of psychosocial factors between peer groups. Trans REMY had higher relative risk of depressive symptoms (ARR 1.7, 95% CI 1.5–2.0), suicidal ideation (ARR 3.2, 95% CI 2.7–3.8), cyberbullying (ARR 2.0, 95% CI 1.6–2.4), and victimization (ARR 1.2, 95% CI 1.1–1.4) compared to cis REMY. Trans REMY had lower relative risk of depressive symptoms compared to trans WY (ARR 0.7, 95% CI 0.6-0.9) but similar risk of suicidal ideation, cyberbullying, and victimization. Moreover, trans REMY reported lower levels of school connectedness compared with cis REMY, but similar levels compared to trans WY.

Table 4 shows analyses assessing associations between absenteeism and psychosocial factors for trans REMY. Depressive symptoms (ARR 2.7, 95% CI 1.3–5.3), suicidal ideation (ARR 3.0, 95% CI 1.5–5.9), and victimization (ARR 2.1, 95% CI 1.0–4.3) were associated with higher relative risk of absenteeism due to mental health concerns. Higher levels of school connectedness among trans REMY were associated with lower relative risk of absenteeism due to mental health concerns (ARR 0.7, 95% CI 0.6–0.9). Cyberbullying was associated with higher relative risk of absenteeism due to harassment (ARR 5.9, 95% CI 1.3–27.2).

## Discussion

To our knowledge, this is the first study to examine absenteeism due to mental health concerns and harassment among trans REMY. Utilizing a large population-based sample permitted us to examine disparities in absenteeism reported by trans REMY compared to peers with whom they share at least one minoritized status and potential experiences of the marginalization targeting those identities. As hypothesized, trans REMY youth reported disparities in absenteeism when compared with some peers and school-based psychosocial factors were associated with their absenteeism.

Supporting our initial hypotheses, we found that trans REMY high school students have elevated risk of absenteeism due to mental health concerns and harassment compared to cis REMY. Counter to our hypotheses, trans REMY had similar risk of each form of absenteeism compared with trans WY. Notably, both trans REMY and trans WY had high rates of absenteeism compared with cis REMY. Similarities in absenteeism between the transgender cohorts may be driven by gender identity-related stigma. Overall, similarities in absenteeism among trans REMY and trans WY and differences in absenteeism between trans REMY and cis REMY may point to the complex interplay of trans REMY’s unique experiences of stigma with absenteeism. Trans REMY who are perceived as different because of gender identity, race, and ethnicity may experience complex stressors, leading to missing school as a maladaptive coping strategy. Further investigation is needed to better understand the role of intersectional stigma in the experiences of trans REMY.

Further consistent with our hypotheses, we identified psychosocial factors associated with forms of absenteeism among trans REMY. School connectedness is a protective factor against absenteeism due to mental health concerns among trans REMY. This finding adds to emerging literature that identifies factors that promote resilience among racially and ethnically minoritized gender expansive youth. For example, a recent qualitative study found that among Black and Latine gender expansive youth, parental gender affirmation is critical for bolstering their resilience, mental health, and self-confidence [23]. Our novel findings point to a source of resilience for trans REMY in another socioecological domain.

We found that risk factors for absenteeism due to mental health concerns among trans REMY included depressive symptoms, suicidal ideation, and victimization. Moreover, cyberbullying was identified as a risk factor among trans REMY for absenteeism due to harassment. To our knowledge, cyberbullying has not been studied in transgender adolescents specifically; however, among transgender adults, qualitative studies have illustrated the harmful effects of cyberbullying [24]. Lesbian, gay, bisexual, transgender, and queer (LGBTQ+) adolescents report higher rates of cyberbullying compared to heterosexual and cisgender peers [24]. Among LGBTQ+ and racially and/or ethnically minoritized youth, rates of cyberbullying are higher than heterosexual peers [25]. Our study shows that cyberbullying is a risk factor for absenteeism, which is particularly salient given the increasing amount of time adolescents spend on social media and the internet [26]. Cyberbullying can occur outside of school grounds and hours and this increasingly pervasive form of harassment can impact a student’s ability to attend school.

Our study has significant implications for school-based supports for trans REMY. Schools that identify chronically absent trans REMY students can address the psychosocial protective and risk factors for absenteeism identified in our study. Counselors and administrators may investigate bullying experienced by these students and encourage them to engage in activities that may help them feel more connected to the school. Pediatricians and other adolescent clinical providers also play an important role in promoting school attendance for trans REMY. They can provide educational workshops and trainings to school staff to enhance awareness of risk factors and protective factors for absenteeism for trans REMY [27]. Furthermore, they may screen trans REMY for absenteeism, and alert schools for youth who are at highest risk so they may be able to act on modifiable factors that can promote school attendance. Our findings also highlight the significant impact cyberbullying can have on absenteeism among trans REMY. While social media and other online platforms can provide a sense of community for adolescents, school counselors, administrators, and pediatricians should screen all adolescents, including those who are gender expansive, for appropriate use of social media and online bullying.

Supporting trans REMY, such as strengthening school connectedness, is especially critical in today’s sociopolitical climate. Schools should be safe places for all young people. However, there are many threats to safe and supportive school environments that perpetuate mistrust among students. This includes recent antitrans legislation involving school bathrooms, participation in sports, requirements for teachers and staff to ‘out’ transgender students, and policies to uphold the use of ‘dead names’ or pronouns of a student’s birth-assigned sex [28]. Moreover, there are ongoing efforts to restrict students from learning about systemic racism [29,30].

With these challenges, additional efforts to support school attendance are even more important. On a systems level, schools and school districts should address systemic discrimination and transphobia targeting trans REMY and other youth. Clinicians also serve as important members and stakeholders within communities who can partner with schools to promote safe and inclusive learning environments. A key step is to improve school climate and recognition and celebration of all forms of diversity. Moving forward, culturally informed interventions to ensure an equitable and inclusive school environment should be explored. In a recent study, the presence of a gender sexuality alliance in schools was associated with fewer mental health symptoms among some gender expansive students [31]. Future studies could investigate how gender sexuality alliances and affinity groups might promote student and school connectedness and celebration of diversity as interventions to reduce school absenteeism. The Gay, Lesbian, and Straight Education Network advocates training school staff in approaches to foster inclusive environments for racially and ethnically minoritized LGBTQ+ youth and promote school connectedness [32,33]. The Centers for Disease Control and Prevention recommend implementation of relationship-building programs [34]. Examples of these programs include sessions focused on teachers strengthening relationships with students, students strengthening relationships with their peers, and events to highlight student experiences [34]. Additionally, policies against both gender identity and sexuality-based harassment as well as trauma-informed policies (particularly around school discipline) should be implemented by school leaders and can be a step toward enhancing school connectedness among trans REMY and other LGBTQ+ youth [34].

Limitations to this study include its observational nature, thus precluding our ability to make causal inferences. Another limitation of this study is birth-assigned sex was not distinguished from gender identity in the survey. Instead, one item queried “Are you transgender?”. Best practices for querying gender identity recommend asking a 2-step question focused on the participant’s current gender identity and birth-assigned sex to inclusively capture individuals who are gender expansive [35–38]. Given this limitation, there is a risk for misclassification bias as youth who identify as gender nonbinary, gender fluid, or gender queer may have not indicated that they are “transgender” on the survey. Furthermore, our study may have limited generalizability to youth who are not sure about or who declined to disclose their gender identity; these young people may be distinct and have different experiences of absenteeism compared to those included in our study. Future studies could explore the distinct experiences of youth who are not sure or who decline to respond to questions about transgender identity. Additionally, this study did not quantify the number of days absent; future studies to explore the number of days absent from school due to mental health concerns or harassment will be crucial because students are at greater risk of poor academic performance and falling behind in school when they miss 15 or more days of school in an academic year [7]. Our focus in this study was to highlight the role minoritized status plays in absenteeism rather than detecting differences between diverse racial and ethnic groups. We would like to recognize that REMY have heterogenous experiences and backgrounds that are not individually captured by our results.

Despite these limitations, our study has several strengths. The data source was a large representative sample and allowed us to make peer group comparisons. Additionally, we uniquely explored two types of absenteeism as follows: due to mental health concerns and due to harassment. The dataset our study utilized contained several psychosocial factors that allowed us to evaluate potential protective and risk factors for school absenteeism. Ultimately, our study highlighted absenteeism and associations among trans REMY within the developmentally critical domain of school. Our findings point to factors that can be bolstered to promote school attendance and aid these resilient youth in better engaging in school and progressing to being educated and thriving adults.

## Figures and Tables

**Table 1:** Demographic characteristics for racially and ethnically minoritized transgender high school students and their peers

	Students, estimated prevalence % (95%CI)^a^			*p*
	Transgender		Cisgender	
	
	REMY (n = 206)	Non-Hispanic White (n = 64)	REMY (n = 24,815)	
Grade				.35
9th	57% (50-64)	48% (35-62)	51% (50-52)	
11th	43% (36-50)	52% (38-65)	49% (48-50)	
Sex				.10
Male	58% (50-66)	46% (34-59)	49% (48-50)	
Female	42% (34-50)	54% (41-66)	51% (50-52)	
Ethnicity				.63^b^
Hispanic/Latino	74% (66-80)	NA	72% (71-73)	
Non-Hispanic	26% (20-34)	100%	28% (27-29)	
Race				.01^b,c^
American Indian or Alaskan Native	7% (4-12)	NA	7% (7-8)	
Asian	11% (6-17)	NA	17% (17-18)	
Black	15% (9-23)	NA	9% (8-9)	
NHOPI	4% (2-10)	NA	2% (2-3)	
White	7% (4-12)^d^	100%	11% (11-12)^d^	
Two or more races	56% (48-64)	NA	53% (52-54)	
Received reduced price or no cost lunch^e^	64% (57-71)	33% (22-46)	65% (64-65)	<.001^c^

CI = Confidence Interval; NHOPI = Native Hawaiian and Other Pacific Islander; REMY = Racially and Ethnically Minoritized. Missing data percentage. Grade 0%, Sex 17.9%, Ethnicity 0.3%, Race 12.5%, Reduced price or no cost lunch 0.6%.

a Estimated prevalence for dichotomous measures and 95% CI are presented with weighted samples.

b Comparison between transgender REMY and cisgender REMY youth.

c *p* value < .05.

d Participants included in REMY column as they identified as Hispanic/Latino.

e These participants answered ‘yes’ to the question: Do you receive free lunch or reduced-price lunch at school?.

**Table 2:** Absenteeism and psychosocial characteristics for racially and ethnically minoritized transgender high school students and their peers

	Students, estimated prevalence % or estimated mean (95% CI)^a^		
	Transgender		Cisgender
	
	REMY (n = 206)	Non-Hispanic White (n = 64)	REMY (n = 24,836)
Absenteeism			
Due to mental health concern	18% (13–25)	23% (15–35)	8% (7–8)
Due to harassment	5% (3–10)	6% (2–19)	0.8% (0.7–0.9)
Depressive symptoms	56% (48–63)	81% (70–89)	34% (33–35)
Suicidal thoughts	44% (37–52)	57% (43–70)	15% (15–16)
Cyberbullied	40% (33–48)	42% (29–57)	21% (21–22)
Victimization	63% (55–70)	77% (64–86)	52% (51–52)
School connectedness^b^ (raw)	14.9 (14.1–15.6)	14.9 (13.6–16.3)	17.3 (17.3–17.4)
School connectedness (standardized)^c^	3.7 (3.5–3.8)	3.7 (3.4–4.0)	4.3 (4.2–4.3)

Missing Data Percentage. Depressive symptoms 0.9%, Suicidal thoughts 0.7%, Cyberbullied 7.5%, Victimization 2.6%, School connectedness 2.8%.

CI = Confidence Interval; REMY = Racially and Ethnically Minoritized youth.

a Estimated prevalence for dichotomous measures or estimated means for continuous measures and 95% CI are presented with weighted samples.

b School connectedness scored by summing together five-items questions each based on a Likert scale 1-5 with a higher score indicating higher levels of school connectedness.

c Standardized measures were obtained by dividing the mean scores by the standard deviation observed in the analytical sample.

**Table 3:** Associations of absenteeism and psychosocial factors among peer groups

Models. Total sample, unadjusted																
	Absenteeism due to mental health		Absenteeism due to harassment		Depressive symptoms		Suicidal thoughts		Cyberbullying		Victimization		School connectedness (raw)^a^		School connectedness (standardized)^a,b^	

	RR (95% CI)	*p*	RR (95% CI)	*P*	RR (95% CI)	*p*	RR (95% CI)	*p*	RR (95% CI)	*p*	RR (95% CI)	*p*	Beta coefficient	*p*	Beta coefficient	*p*
Peer group comparisons																
Trans REMY versus trans WY^c^	0.8 (0.5–1.4)	.40	0.9 (0.2–3.6)	.87	0.7 (0.6–0.8)	<.001^d^	0.8 (0.6–1.0)	.10	1.0 (0.7–1.4)	.83	0.8 (0.7–1.0)	.03^d^	−0.10 (−1.6 to 1.4)	.91	−0.02 (−0.4 to 0.4)	.91
Trans REMY versus cis REMY^c^	2.4 (1.8–3.3)	<.001^d^	6.7 (3.4–13.3)	<.001^d^	1.6 (1.4–1.9)	<.001^d^	2.9 (2.5–3.5)	<.001^d^	1.9 (1.6–2.3)	.001^d^	1.2 (1.1–1.3)	.001^d^	−2.5 (−3.2 to −1.8)	<.001^d^	−0.6 (−0.8 to −0.4)	<.001^d^
Models. Total sample, adjusted^e^																
	Absenteeism due to mental health		Absenteeism due to harassment		Depressive symptoms		Suicidal thoughts		Cyberbullying		Victimization		School connectedness (raw)^a^		School connectedness (standardized)^a,b^	

	ARR (95% CI)	*p*	ARR (95% CI)	*P*	ARR (95% CI)	*p*	ARR (95% CI)	*p*	ARR (95% CI)	*p*	ARR (95% CI)	*p*	Beta coefficient	*p*	Beta coefficient	*p*

Peer group comparisons																
Trans REMY versus trans WY^c^	0.7 (0.4–1.2)	.26	2.1 (0.4–10.0)	.35	0.7 (0.6–0.9)	.01^d^	0.9 (0.6–1.1)	.28	1.2 (0.8–1.7)	.44	0.8 (0.7–1.0)	.10	−0.9 (−2.2 to 0.4)	.17	−0.2 (−0.5 to 0.10)	.17
Trans REMY versus cis REMY^c^	2.9 (2.1–4.0)	<.001^d^	8.1 (4.0–16.6)	<.001^d^	1.7 (1.5–2.0)	<.001^d^	3.2 (2.7–3.8)	<.001^d^	2.0 (1.6–2.4)	<.001^d^	1.2 (1.1–1.4)	.01^d^	−2.5 (−3.2 to −1.7)	<.001^d^	−0.6 (−0.8 to −0.4)	<.001^d^

Models represent the abbreviated output from Poisson regression models.

ARR = adjusted relative risk; CI = confidence interval; Cis REMY = racially and ethnically minoritized cisgender youth; RR = relative risk; Trans REMY = racially and ethnically minoritized transgender youth; Trans WY = White transgender youth.

a Results are from linear regression analyses for school connectedness.

b Standardized measures were obtained by dividing the mean scores by the standard deviation observed in the analytical sample.

c Referent group.

d *p* < .05.

e Models adjusted for sex assigned at birth, grade, and socioeconomic status (i.e., receipt of free/reduced cost lunch).

**Table 4:** Associations of psychosocial factors with absenteeism in racially and ethnically minoritized transgender high school students

	Relative risk (95% CI)			
	Absenteeism due to mental health (unadjusted)^a^	Absenteeism due to mental health (adjusted)^b^	Absenteeism due to harassment (unadjusted)^a^	Absenteeism due to harassment (adjusted)^b^
Depressive symptoms	2.7 (1.2-5.7)^c^	2.7 (1.3-5.3)^c^	0.8 (0.2–3.0)	0.6 (0.2–1.8)
Suicidal thoughts	3.5 (1.7-7.0)^c^	3.0 (1.5-5.9)^c^	2.2 (0.6–8.0)	1.1 (0.3–3.4)
Cyberbullied	1.3 (0.7–2.3)	1.2 (0.7–2.2)	6.5 (1.4-30.8)^c^	5.9 (1.3-27.2)^c^
Victimization	2.1 (1.0–4.5)	2.1 (1.0-4.3)^c^	2.3 (0.5–11.5)	2.2 (0.5–9.0)
School connectedness (raw)	0.9 (0.9-1.0)^c^	0.9 (0.9-1.0)^c^	0.9 (0.8–1.0)	0.9 (0.8–1.1)
School connectedness (standardized)^d^	0.6 (0.5-0.8)^c^	0.7 (0.6-0.9)^c^	0.67 (0.4–1.1)	0.7 (0.4–1.1)

CI = Confidence Interval.

a Unadjusted relative risk obtained from Poisson regression analyses assessing associations between the two forms of absenteeism and psychosocial factors in racially and ethnically minoritized transgender high school students.

b Poisson regression adjusted for sex, socioeconomic status (receipt of free/reduced-cost lunch), and grade.

c *p* < .05.

d Standardized measures were obtained by dividing the mean scores by the standard deviation observed in the analytical sample.
